# Pleural Involvement in IgG4-Related Disease: Case Report and Review of the Literature

**DOI:** 10.3390/diagnostics11122177

**Published:** 2021-11-23

**Authors:** Federico Mei, Massimiliano Mancini, Giulio Maurizi, Andrea Vecchione, Lina Zuccatosta, Erino Angelo Rendina, Stefano Gasparini

**Affiliations:** 1 Department of Biomedical Sciences and Public Health, Università Politecnica delle Marche, 60126 Ancona, Italy; lina.zuccatosta@ospedaliriuniti.marche.it (L.Z.); s.gasparini@univpm.it (S.G.); 2 Respiratory Diseases Unit, Azienda Ospedaliero-Universitaria “Ospedali Riuniti”, 60126 Ancona, Italy; 3 Morphological and Molecolar Pathology Unit, Ospedale Sant’Andrea, 00189 Rome, Italy; mamancini@ospedalesantandrea.it (M.M.); andrea.vecchione@uniroma1.it (A.V.); 4 Thoracic Surgery Unit, Ospedale Sant’Andrea, 00189 Rome, Italy; giulio.maurizi@uniroma1.it (G.M.); erinoangelo.rendina@uniroma1.it (E.A.R.)

**Keywords:** IgG4-related disease, pleural disease, pleural effusion, pleural fluid analysis, image-guided pleural biopsies, thoracoscopy, thoracic ultrasound

## Abstract

Diagnostic work-up of IgG4-related disease (IgG4-RD) pleural involvement is a complex task, as there is a broad spectrum of differential diagnoses to consider. We report the case of a patient presenting with relapsing pleural effusion, discussing the main challenges for achievement of a definite diagnosis. A 63-year-old man was admitted for pleural effusion prevalent on the ride side, initially labeled as idiopathic non-specific pleuritis, based on tissue evaluation after a medical thoracoscopy. He was started on steroids with initial improvement, but a later CT scan showed a relapse of pleural effusion associated with diffuse pleural thickening; a subsequent surgical pleural biopsy revealed features suggestive for IgG4-RD, with a marked increase of IgG4 positive plasma cells. High IgG4 serum levels were also found. The present case underlines the importance of increasing awareness of this potential condition among physicians in order to properly guide the diagnostic work-up, as it is likely that IgG4-RD accounts for a proportion of patients with pleural effusions, labeled as idiopathic. In particular, in patients with unexplained pleural effusion, IgG4-RD should be included among differential diagnoses when lymphoplasmacytic infiltration is observed, and a multidisciplinary interaction between clinicians and pathologists appears crucial for an accurate diagnosis and an appropriate management.

## 1. Introduction

IgG4-related disease (IgG4-RD) is a chronic, systemic fibro-inflammatory disorder characterized by lymphoplasmacytic infiltration rich in IgG4-positive plasma cells, storiform fibrosis, obliterative phlebitis and often, but not always, elevated serum IgG4 levels [1,2].

IgG4-RD, firstly described in the pancreas, can virtually affect any organ, including kidneys, retroperitoneum, aorta, lymph nodes, skin, as well as lungs and pleura. Thoracic involvement occurs in up to 50% of patients with heterogenic features, such as mediastinal lymphadenopathy, lung consolidations, pericarditis and pleural manifestations [3,4]. In particular, prevalence of pleural effusion and thickening ranges from 5% to 16% [5,6,7,8]. In most of cases, IgG4-RD pleural fluid is consistent with exudate, and it is characterized by lymphocytes, plasma cells and high IgG4 concentration. Accordingly, histopathological examination of pleural biopsy reveals dense fibrosing pleuritis accompanied by prominent lymphoplasmacytic inflammation, including IgG4-positive plasma cells [9,10]. 

Diagnostic work-up of IgG4-RD thoracic involvement may be challenging, as there is a broad spectrum of differential diagnoses to consider, including multisystemic diseases and conditions with similar clinical-radiological appearance or with elevated IgG4 tissue levels.

Therefore, a multidisciplinary interaction between clinicians from different specialties and pathologists appears essential for an accurate diagnosis. In this context, the role of interventional pulmonologists and thoracic surgeons is crucial to obtain adequate pleural samples, in order to allow pathologists to detect the peculiar histological features, often unevenly distributed.

Herein, we report the case of a patient presenting with relapsing pleural effusion and we discuss the main challenges of this complex diagnostic work-up.

## 2. Case Presentation 

A 63-year-old man was admitted to our Respiratory Disease Unit at the University Hospital—Ancona, for a 6-month exertional dyspnea and bilateral pleural effusion prevalent on the ride side, detected on chest computed tomography (CT).

He was former smoker without occupational exposure to asbestos. His medical history was remarkable for asymptomatic brain aneurysm, blood hypertension, multiple lumbar disc herniation. On admission to our unit, physical examination, oxygen saturation on room air, heart rate and blood pressure were normal, whilst breathing sound was suppressed at the third right lower lung fields.

**Figure 1:**
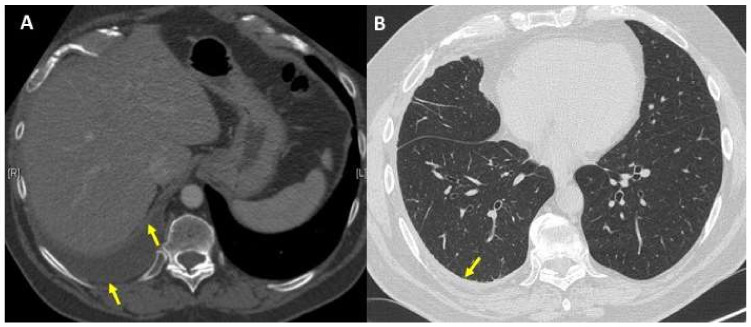
(**A**) Chest computed tomography (CT) scan showed a moderate right pleural effusion with parietal and visceral pleural thickening (yellow arrows); (**B**) normal lung parenchyma without significant abnormalities; concomitant parietal pleural thickening (yellow arrow).

**Figure 2:**
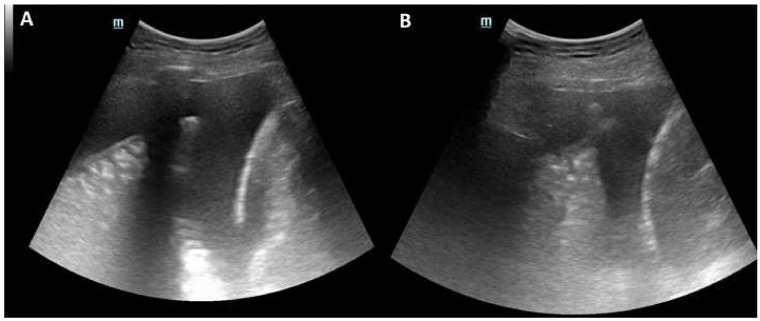
(**A**) Thoracic ultrasound (TUS) showed a moderate right hyperechogenic pleural fluid; (**B**) Atelectasis of right lower lobe.

The patient first underwent a repeated CT scan that allowed us to rule out a pulmonary embolism and confirmed moderate right pleural effusion with parietal and visceral pleural thickening, in the absence of significant parenchymal abnormalities (Figure 1). Thoracic ultrasound (TUS) revealed hyperechogenic pleural fluid with atelectasis of basal segments of the right lower lobe (Figure 2); at thoracentesis, fluid appeared cloudy and yellow coloured, and a physico-chemical exam was consistent with exudate and microbiological tests, including an acid-alcohol-fast bacilli (AAFB) search, were negative (Table 1).

**Figure 3:**
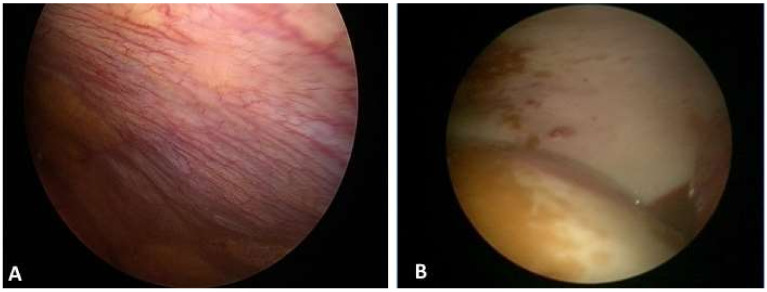
Thoracoscopic findings: (**A**) parietal pleura hyperemia; (**B**) with fibrotic plaques.

A subsequent medical thoracoscopy (MT) revealed the presence of yellow pleural fluid (overall 1800 mL removed) and parietal pleura hyperemia with fibrotic plaques (Figure 3). Ten pleural biopsies were obtained by forceps on parietal pleura and histopathological examination documented a large lymphoplasmacytic infiltration, fibrosis, reactive mesothelial cells and vascular proliferation, in absence of neoplastic lesions or granulomas; the final diagnosis was suggestive for non-specific pleuritis (NSP).

An extensive diagnostic work-up, including echocardiogram, abdominal angiography CT scan, autoimmune, viral, and bacterial serology, failed to detect any potential known cause of NSP and blood tests were normal, except for a mild elevation of C-reactive protein. Thus, the patient was diagnosed with idiopathic NSP and therapy was started with steroids (Methylprednisolone 0.5 mg/kg, tapered in one month with clinical and radiological improvement. 

**Figure 4:**
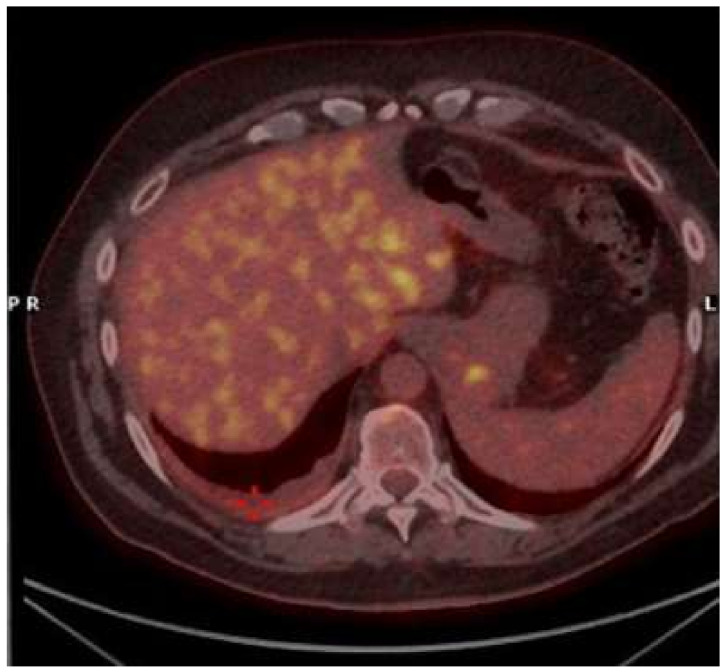
Positron emission tomography-fluorodeoxyglucose (PET-FDG) showed small amount of right pleural effusion associated with diffuse pleural thickening, resulted as slightly absorbing (red cross).

Six months later, the patient complained chest discomfort and mild dyspnea, and CT scan showed a relapse of small amount of right pleural effusion associated with diffuse pleural thickening; a PET-FDG showed slight captation on right basal parietal pleural without abnormal captations in other organs (Figure 4).

**Figure 5:**
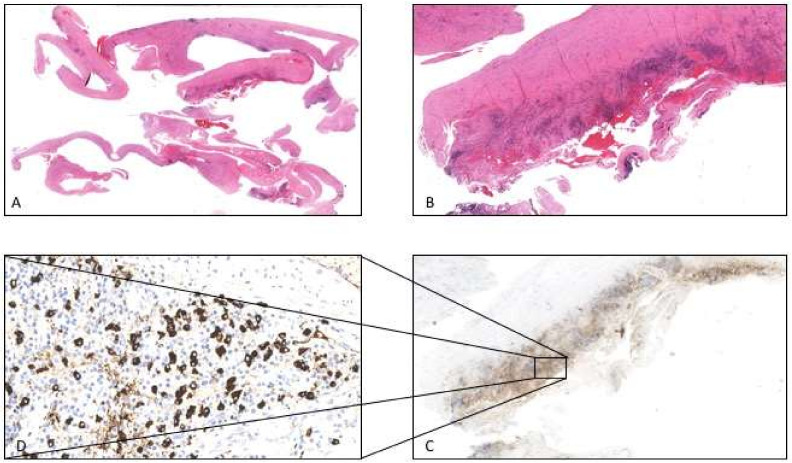
(**A**) Histologic examination showed diffuse fibrosing pleuritis, with occasional hyaline features, fibrinous exudate (1× original magnification, EE stain). (**B**) Dense patchy lymphoid infiltrates rich in plasma cells could be observed (10× original magnification EE stain). (**C**) Immunoperoxidase stain revealed the lymphoid infiltrates to be composed of numerous IgG4 positive plasma cells (10× original magnification Immunoperoxidase stain). (**D**) At greater magnification plasma cells quantification showed up to 50 IgG4+/HPF) with an IgG4^+^/IgG^+^ ratio > 40% (40× original magnification Immunoperoxidase stain).

Due to the limited pleural effusion and the absence of sliding at TUS, we decided not to repeat thoracoscopy, but to refer the patient to the Thoracic Surgery Unit of Sant’ Andrea University Hospital (Rome) for a surgical pleural biopsy. Histological examination revealed a diffuse fibrosing pleuritis, with occasional hyaline features, fibrinous exudate, and a dense lymphocytic and plasma cell inflammation. Interestingly, inflammatory infiltrate was diffuse but with irregular distribution throughout the histological specimen. Immunoperoxidase stains showed a marked increase of IgG4 positive plasma cells (up to 50/HPF) with an IgG4^+^/IgG^+^ ratio >40% reaching the diagnostic threshold level for IgG4 disease [9]. High IgG4 serum levels were found (324 mg/dL) [11]. Based on these findings, the patient was diagnosed with IgG4-related pleuritis (Figure 5).

After exclusion of systemic involvement of IgG4 disease, steroid therapy was started (Prednisone 0.5 mg/kg/d for 2 weeks, tapering up to 2.5 mg/day as a maintenance dose for an overall period of 6 months) with a complete and stable resolution of pleural effusion, improvement of respiratory symptoms and a progressive reduction of IgG4 serum levels, returned within normal limits (64 mg/dL).

## 3. Discussion

The present case report describes a relatively uncommon cause of relapsing exudative monolateral pleuritis, finally diagnosed with IgG4-RD, but initially labeled as idiopathic NSP, underlining the challenging diagnostic work-up of this condition. 

IgG4-RD is an increasingly recognized fibro-inflammatory disorder, firstly described in association with autoimmune pancreatitis, salivary and lacrimal glands inflammation, formerly known as Mikulicz’s disease [1]. Clinical manifestations are quite pleomorphic, since IgG4-RD can potentially involve any organ either synchronously or asynchronously, and morphological alterations may be different at intra-organ level (Table 2) [2].

Thoracic involvement, indeed, includes interstitial lung disease, inflammatory pseudotumours, fibrosing mediastinitis, lymphadenopathy and pleural disease [3,4]. With reference to pleural involvement, nodules and thickening are observed in 16% of cohorts, whilst pleural effusion is less frequent, being described in nearly 5% of cases [5,6]. However, pleural effusion is commonly faced by clinicians in daily practice, as it may occur in several conditions with different prognoses, as well as treatment options [12]. Of note, the increasing burden of pleural diseases in the last decades has coupled with an outstanding evolution in medical technologies related to diagnosis and management of such conditions, no longer reserved only to thoracic surgeons or interventional radiologists, but now widely available in interventional pulmonology centers, leading also to the development of a less invasive, but accurate approach, such as TUS-guided percutaneous pleural biopsy [13,14]. Therefore, it is important to increase awareness of this potential cause among physicians from different specialties in order to properly guide the diagnostic work-up, as it is likely that IgG4-RD could account for a proportion of patients with pleural effusions of idiopathic cause [15,16,17]. The prevalence of IgG4-RD, diagnosed during follow-up in subjects with pleural effusions previously labeled as idiopathic, was investigated in two recent studies, both reporting a proportion around 35% [18,19]. In detail, in the study by Murata and colleagues [19], 12 of 35 (34%) patients with undiagnosed pleural effusions were found to have marked IgG4-positive plasma cell infiltration in the pleura by IgG4 immunostaining and elevated effusion IgG4 concentrations, while Kasashima et al. [18] reported that eight out of 22 (36%) patients with fibroinflammatory pleural lesions of idiopathic cause met defined diagnostic criteria for IgG4-RD. Based on international consensus histopathology criteria [9], the achievement of a high confidence final diagnosis of IgG4-RD relies on a combination of clinical, biochemical, radiological and histological features, and no single test is diagnostic [20]. First, a thorough clinical history focused on extrathoracic signs and symptoms, a careful physical examination and pleural fluid analysis are key elements to determine the subsequent diagnostic steps.

Overall, IgG4-RD pleural disease occurs more frequently in men (78%), the mean age is 63.5 ± 14.7 years old and an exudative pattern is reported in almost all cases [8]. Pleural effusion cytometry is characterized by predominance of lymphocytes and plasma cells and a high concentration of IgG4 is identified in some, but not all cases. The potential added value of pleural fluid cell block examination in this context has been investigated by Kasashima and colleagues [18]. Cell block examination, allowing IgG4 immunostaining, has been shown to be helpful in the diagnosis of IgG4-RD pleuritis, and in particular, a positive correlation of the number of IgG4+ cells and eosinophils in a pleural tissue examination was observed. Therefore, in the case of high pre-test probability of IgG4-RD pleural disease, the cell block of pleural fluid should be sent for specific immunohistochemistry/staining. Interestingly, high levels of adenosine deaminase (ADA) in pleural fluid (>40 U/L) have been described in a significant number of cases of IgG4-RD pleuritis, although in our patient they were below normal limits. ADA is a hallmark of lymphocytes activation and is widely used in the auxiliary diagnosis of tuberculous pleuritic. As a result, after ruling out mycobacterial infection using proper tools, elevated ADA levels in pleural fluid may be useful for identifying IgG4-RD pleuritis [21]. 

With reference to serology, it is important to underline that high IgG4 levels (>135 mg/dL), significantly contribute to the achievement of a definite diagnosis in a suggestive context, but their absence does not allow us to exclude this condition. IgG4 levels within normal limits were detected in approximately 50% of biopsy-proven, clinically active IgG4-RD patients, while, on the contrary, high concentrations were found in 5% of healthy subjects [6]. These data raise question about the exact pathogenic role of IgG4 antibodies, more likely to represent a marker of an immune-mediated process, as part of a more complex inflammatory disease. In fact, inflammatory markers, such as C-reactive protein and LDH, may be also elevated in some cases, as they were in our case.

Therefore, in this context, histopathological assessment plays a crucial role in order to improve the diagnostic confidence, as differential diagnosis ranges from other multisystem diseases (e.g., sarcoidosis, connective tissue disease), to those with similar clinico-radiological features (e.g., interstitial lung diseases, cancer, lymphoma) or to conditions with local and systemic inflammation with elevated IgG4 tissue levels (e.g., ANCA-associated vasculitis, multicentric Castleman’s disease) [22]. Furthermore, the response of a majority of the aforementioned diseases to corticosteroid therapy can represent another confounding factor in achieving the correct diagnosis of IgG4-RD. Several techniques to obtain pleural tissue are currently available, including TUS- or CT-guided pleural biopsy, MT, video-assisted thoracoscopic (VATS) and open surgical biopsy [13,14], but in the case of unexplained pleural effusion without significant diffuse thickening, MT is the preferred approach.

Thoracoscopic findings at endoscopic view are usually not specific, including pleural hyperemia, pleural thickening, hyalinized white plaques, expression of collagen fibers deposit and small nodular lesions [23]. The main histopathological features are dense lymphoplasmacytic infiltrate, obliterative phlebitis, storiform fibrosis, more than 10 IgG4-positive plasma cells per high-power field and an IgG4/IgG-positive plasma cell ratio of more than 40%; a moderate number of eosinophils granulocytes can also be observed. Interestingly, pathological findings in the lungs are slightly different from those reported in other organs in IgG4-RD, as storiform fibrosis or obliterative phlebitis are less frequently observed. The thresholds for the absolute number of IgG4+ plasma cells per high-power field varies from organ to organ and to some extent by the methodology of tissue sampling being usually higher in surgical biopsies compared to non-surgical ones [24]. The presence of epithelioid cell granuloma, prominent neutrophilic infiltrate, abscess and necrosis is not suggestive of IgG4-RD and should raise suspicions of other conditions.

IgG4-RD lung involvement may occur either in isolation or as part of a multisystem process, and, due to its significant prevalence, all patients with an established diagnosis of extra thoracic disease should undergo proper chest imaging. In an elegant study by Corcoran and colleagues [4], chest CT and/or X-rays of patients without symptomatic pulmonary manifestations of IgG4-RD, but proven extra-thoracic disease, were reviewed, with occult thoracic abnormalities found in almost half of the cohort, including three cases with pleural effusion/thickening.

Therefore, an accurate assessment of any potential organ involvement is essential for a proper management, in order to avoid irreversible damages. Steroid therapy remains the first line approach, with dosing varying according to local practice, but 0.5–1 mg/kg daily of oral prednisone is commonly used. Assuming a favorable response, this can be gradually tapered over 3–6 months. In resistant/relapsing cases, additional immunomodulatory medications such as rituximab, azathioprine and mycophenolate can be used, although there is no robust evidence to guide treatment choice in this field [25].

## 4. Conclusions

Pleural involvement in IgG4-RD can occur in isolation, or association with pulmonary or extrapulmonary manifestations, and its prevalence appears to increase over time, althougth it remains an under-recognized condition. It is important to increase awareness of this potential condition and its varying manifestations among physicians from different specialties in order to properly guide the diagnostic work-up, as it is likely that IgG4-RD accounts for a proportion of patients with pleural effusions labeled as idiopathic. In particular, in patients with unexplained pleural effusion, IgG4-RD should be included among differential diagnoses when lymphoplasmacytic infiltration is observed, and a multidisciplinary interaction between clinicians and pathologists appears crucial for an accurate diagnosis and appropriate management. Further studies on large cohorts are needed to assess true prevalence, clinical evolution, and response to treatment of pleural IgG4-RD.

## Figures and Tables

**Table 1:** Pleural fluid characteristics.

Parameter	Results
Appearance	Cloudy
Colour	Yellow
Total protein (g/dL)	4.5 g/dL
Cholesterol (mg/dL)	77 mg/dL
Lactate dehydrogenase—LDH (U/liter)	515 U/L
Glucose (mg/dL)	105 mg/dL
White cells count	2730/mcL (56% of mononucleated cells)
Cytology	Reactive mesothelial cells and lymphocytes
Microbiology	Negative
AAFB	Negative

**Table 2:** IgG4-related disease (IgG4-RD): summary of clinical manifestations by organ system.

Organ	Clinical and Radiological Findings	Differential Diagnosis
*Orbital and peri-orbital area*	Orbital pseudotumor; dacryoadenitis; dacrocystitis; Orbital mass lesions	Lymphoma; Graves’disease; Granulomatosis with polyangiitis (Wegener’s); Sarcoidosis
*Meninges*	Hypertrophic pachimeningitis	Inflammatory myofibroblastic tumor; Granulomatosis with polyangiitis (Wegener’s); Giant cell arteritis; Langerhans cell histiocytosis; Sarcoidosis
*Pituitary gland*	Hypophysitis	Neoplasms; Histiocytosis; Hypophysitis: Primary or Secondary (sarcoidosis, ipilimumb-induced)
*Salivary glands*	Sialoadenitis (Mikulicz Disease)	Lymphoma; Sjögren’s syndrome; Sarcoidosis; Sialodocholithiasis
*Upper airways*	Nasal polyps, allergic rhinitis, nasal obstruction, rhinorrhea, anosmia, chronic sinusitis, eosinophiic angiocentric fibrosis	Allergic disease; Churg-Strauss syndrome; Granulomatosis with polyangiitis (Wegener’s); Sarcoma
*Thyroid gland*	Hypothyroidism; thyroid gland enlargement; Riedel’s thyroiditis	Thyroid lymphoma; Differentiated thyroid carcinoma; Other malignancy
*Cardiovascular system*	Constrictive pericarditis; peri-aortitis; inflammatory aneurysm; coronary arteritis	
*Respiratory system*	Parenchymal lung consolidations, inflammatory pseudotumor, central airway disease, interstitial lung disease, pleural effusion, pleural thickening, pleural nodules or masses	Malignancy; Inflammatory myofibroblastic tumor; Sarcoidosis; Castleman’s disease; Lymphomatoid disease; Pleural malignancy; Non specific pleuritis; Interstitial lung disease
*Mediastinum and Retroperitoneum*	Lymphoadenopathy; retroperitoneum fibrosis	Sarcoidosis; Castleman’s disease; Lymphomatoid disease
*Gastrointestinal system*	Autoimmune pancreatitis; Inflammatory mesenteritis; Sclerosing cholangitis; Mass lesions liver	Pancreatic cancer; Cholangiocarcinoma; Primary sclerosing cholangitis; Hepatocarcinoma
*Haematopoietic-Lymphatic system*	Lymphoadenopaty; Eosinofilia; Polyclonal hypergammaglobulinemia	Myeloma; Lymphomatoid and Myeloid disease
*Urinary system*	Tubulointerstitial nephritis; Tumoral lesions	Lymphoma; Renal cell carcinoma; Paucimmune Necrotizing Glomerulonephritis; Sarcoidosis Sjögren’s syndrome Systemic lupus erythematosus

## Data Availability

The data presented in this study are available on request from the corresponding author. The data are not publicly available due to the privacy policy of the centers involved in the study.
